# Human Papillomavirus Vaccination Strategies

**DOI:** 10.3201/eid1312.070701

**Published:** 2007-12

**Authors:** Santiago Pérez Cachafeiro

**Affiliations:** * Galician Agency for Health Technology Assessment, Santiago de Compostela, Spain

**Keywords:** HPV vaccines, cost-benefit analysis, health policy, letter

**To the Editor:** An article by Elbasha et al. in the January 2007 issue of Emerging Infectious Diseases showed an economic evaluation of human papillomavirus (HPV) vaccination strategies (*1*). In this model, incremental cost-effectiveness ratio (ICER) calculations were based on costs measured as US dollars for 2005 and effectiveness measured as quality-adjusted life years (QALYs). Authors presented these data transparently and showed costs and QALYs of each strategy in 2 tables, where they did not show ICER of dominated options; i.e., “Strategy A is dominated if there is another strategy, B, that is more effective and less costly than strategy A” (*1*). Unfortunately, splitting data into 2 tables can be misleading.

First, ICERs of strategies for vaccination at the age of 12 (70% coverage) compared with a strategy of no vaccination showed that the strategy of vaccinating 12-year-old girls and boys is dominated by other strategies. Furthermore, vaccination of 12-year-old girls only and vaccination of 12-year-old girls only with catch-up (vaccination of girls and women 12–24 years of age) have lower ICERs, which could be interpreted as the most cost-effective approaches.

Finally, ICERs of strategies of vaccinating at 15 and 18 years of age (50% coverage) are presented without comparison strategies. Thus, one might assume that these strategies are compared with the baseline strategy (vaccination of 12-year-old girls only); however, they are compared with the no-vaccination strategy.

The transparency of the Elbasha et al. article enabled us to build a new table based on their data (Table). In our table, ICERs of the whole set of strategies showed that vaccination of 12-year-old girls only is dominated by the vaccination of 18-year-old women plus a catch-up strategy (women 18–24 years of age), although older groups have lower coverages.

**Table:** Cost-effectiveness analysis of alternative human papillomavirus vaccination strategies*

Strategy	Discounted			Incremental†		
	Cost	QALY		Cost	QALY	ICER ($/QALY)‡
No vaccination	$72,659,302	2,698,711		–	–	–
12-y-old girls	$74,042,990	2,699,178		$1,383.688	467	Dominated
18-y-old women + 18–24-y-old female catch-up	$73,553,847	2,699,192		$894,545	481	$1,860
15-y-old girls + 15–24-y-old female catch-up	$73,895,046	2,699,214		$341,199	22	$15,509
12-y-old girls and boys	$78,707,825	2,699,327		$4,812,779	113	Dominated
12-y-old girls + 12–24-y-old female catch-up	$74,815,667	2,699,343		$920,621	129	$7,137
18-y-old women and men + 18–24-y-old female and male catch-up	$77,535,383	2,699,385		$2,719,716	42	$64,755
15-y-old girls and boys + 15–24-y-old female and male catch-up	$78,455,750	2,699,404		$920,367	19	$48,440
12-y-old girls and boys + 12–24-y-old female catch-up	$79,746,357	2,699,461		$1,290,607	57	$22,642
12-y-old girls and boys + 12–24-y-old female and male catch-up	$81,761,210	2,699,506		$2,014,853	45	$44,775

*QALY, quality-adjusted life year; ICER, incremental cost-effectiveness ratio; $, US dollars. †Based on discounted costs reported by Elbasha et al. (*1*). ‡Compared with the preceding nondominated strategy. Strategy A is dominated if there exists another strategy, B, that is more effective and less costly than strategy A.

In addition, I point out 2 particulars. First, epidemiology of HPV varies between countries (*2*), probably because of differences in culture and sexual habits. Thus, vaccination at older ages should be considered in countries in which prevalence of adolescent sexual activity or HPV is low. Second, higher vaccine coverage in older groups would decrease ICERs of these strategies (*1*). Both facts could reflect the real situation in some countries, e.g., Spain (*2**,**3*).

In conclusion, economic evaluations of HPV vaccination strategies should have broader sensitivity analysis to include as many country-specific realities as possible. To avoid misunderstandings that could lead policymakers to misallocate funds, these results should be evident to readers.
